# Fabrication of Quercetin-Functionalized Morpholine and Pyridine Motifs-Laden Silk Fibroin Nanofibers for Effective Wound Healing in Preclinical Study

**DOI:** 10.3390/pharmaceutics16040462

**Published:** 2024-03-26

**Authors:** Govindaraj Sabarees, Vadivel Velmurugan, Siddan Gouthaman, Viswas Raja Solomon, Subramani Kandhasamy

**Affiliations:** 1 Department of Pharmaceutical Chemistry, SRM College of Pharmacy, SRM Institute of Science and Technology, Kattankulathur 603203, India; sabareesgovind@gmail.com; 2 Organic Material Laboratory, Department of Chemistry, Indian Institute of Technology, Roorkee 247667, India; goureshwran@gmail.com; 3 Medicinal Chemistry Research Laboratory, MNR College of Pharmacy, Gr. Hyderabad, Sangareddy 502294, India; vrajasolomon@gmail.com; 4 School of Mechanical and Electrical Engineering, Quanzhou University of Information Engineering, Quanzhou 362000, China

**Keywords:** spun silk fibroin, functionalized quercetin, antioxidant, antibacterial, tissue engineering, rapid wound healing

## Abstract

Choosing suitable wound dressings is crucial for effective wound healing. Spun scaffolds with bioactive molecule functionalization are gaining attention as a promising approach to expedite tissue repair and regeneration. Here, we present the synthesis of novel multifunctional quercetin with morpholine and pyridine functional motifs (QFM) embedded in silk fibroin (SF)-spun fibers (SF-QFM) for preclinical skin repair therapies. The verification of the novel QFM structural arrangement was characterized using ATR-FTIR, NMR, and ESI-MS spectroscopy analysis. Extensive characterization of the spun SF-QFM fibrous mats revealed their excellent antibacterial and antioxidant properties, biocompatibility, biodegradability, and remarkable mechanical and controlled drug release capabilities. SF-QFM mats were studied for drug release in pH 7.4 PBS over 72 h. The QFM-controlled release is mainly driven by diffusion and follows Fickian’s law. Significant QFM release (40%) occurred within the first 6 h, with a total release of 79% at the end of 72 h, which is considered beneficial in effectively reducing bacterial load and helping expedite the healing process. Interestingly, the SF-QFM-spun mat demonstrated significantly improved NIH 3T3 cell proliferation and migration compared to the pure SF mat, as evidenced by the complete migration of NIH 3T3 cells within 24 h in the scratch assay. Furthermore, the in vivo outcome of SF-QFM was demonstrated by the regeneration of fresh fibroblasts and the realignment of collagen fibers deposition at 9 days post-operation in a preclinical rat full-thickness skin defect model. Our findings collectively indicate that the SF-QFM electrospun nanofiber scaffolds hold significant capability as a cost-effective and efficient bioactive spun architecture for use in wound healing applications.

## 1. Introduction

The skin is essential in protecting the body from external harm. Any injury or damage inflicted upon living skin or tissue can be classified as a wound. There are different types of wounds, such as bruises, incisions, and cuts, depending on the nature of the injury. Various internal and external factors such as local blood supply disorders, heat, chemicals, electrical current, external force, and surgery can cause adverse conditions that trigger diverse physiological responses within the body [1]. Inadequate wound care can lead to complications such as bleeding, infection, inflammation, scarring, and impaired angiogenesis and regeneration [2,3]. Therefore, it is crucial to dress wounds promptly to protect the wound bed and expedite wound healing by enhancing potent regeneration without adverse effects. Although numerous synthetic wound dressings have been functionalized and manufactured using different polymers and bioingredients, their physicochemical and biological qualities are limited. This limitation poses a significant challenge in enhancing their multifunctionality, such as improving resistance to invading infections, eradicating potent free radicals, and ensuring excellent biocompatibility simultaneously for rapid wound healing [4].

Despite the limitations mentioned earlier, recent advances in synthetic bioactive compounds have shown promising results in tissue engineering and therapeutic applications [5]. Aromatic system-functionalized synthetic and natural polymer biomaterials with abundant electronegative atoms have been found to have multiple roles in these applications. One interesting finding is that regenerative biopolymers containing multifunctional groups with bioactive moieties have the potential to reduce inflammation, protect against oxidative damage, and prevent microbial infections. This helps promote faster and more efficient wound healing without forming scar tissue [6]. Quercetin, a natural compound found in fruits, vegetables, and herbs, has shown great promise in wound healing [7]. It has antimicrobial properties, inhibiting the growth of bacteria, viruses, and fungi [8]. Additionally, it acts as a powerful antioxidant, protecting cells from oxidative damage and enhancing the body’s defense mechanisms. Flavonoids containing quercetin stimulate the production of growth factors and promote angiogenesis, thereby improving blood supply to the wound and facilitating better tissue regeneration [9].

Morpholine is a bioactive agent commonly used in antiseptic solutions and ointments for wound care. This chemical compound has potent antimicrobial properties that inhibit bacterial growth and prevent infection, making it a vital asset in wound treatment [10,11]. When applied topically, morpholine is highly effective in eliminating or restraining the growth of harmful microorganisms, which reduces the likelihood of complications and speeds up the healing process [12,13]. On the other hand, pyridine is another chemical compound that is effective in wound healing due to its unique properties [14,15]. Its chemical structure allows for easy penetration through membranes, facilitating direct interaction with vital cellular components involved in healing [16]. Pyridine helps to reduce inflammation by modulating the release of pro-inflammatory mediators and promoting the migration and proliferation of keratinocytes [17]. Pyridine-based formulations are available in various forms and have gained attention for their efficacy and safety in wound healing. They are particularly useful in managing acute and chronic wounds like ulcers, burns, and surgical incisions [18].

Silk fibroin (SF) is a material derived from *Bombyx mori* and has gained significant interest due to its unique properties. These properties include customizable mechanical strength, biodegradability, non-allergenicity, non-toxicity, biocompatibility, hemostatic properties, and processability without chemical cross-linkers [19,20]. SF can be processed into various formats, such as sponges, nanofibers, hydrogels, particles, gauze, wafers, and films, making it a viable option for wound healing applications [21]. Electrospinning is a widely recognized and effective approach for producing constructs in the form of non-woven fiber networks, which offers significant advantages in generating fibrous membranes that are loose, porous, and exhibit an increased surface-to-mass ratio [22,23]. SF is a promising option for various biomedical applications, including tissue engineering, developing hemostatic mats, antibacterial meshes, wound dressing, drug delivery, and 3D implants [24,25]. Combining silk fibroin electrospun fibrous materials with robust bioactive molecules has brought about a profound transformation in biomedical research. Such combinations offer numerous advantages, including heightened bioactive efficacy, therapeutic attributes, and functional capabilities. Notably, their capacity to expedite and facilitate comprehensive healing, particularly in patients with infected wounds, holds substantial implications for enhancing patient outcomes and minimizing recovery time [26].

The purpose of this study is to create and test a dressing made from silk fibroin fibers that contain quercetin, a compound with multiple healing properties. The dressing will have morpholine and pyridine functional motifs, which will enhance its effectiveness. The dressing will be used to treat skin wounds. The research will involve designing, developing, characterizing, and evaluating the dressing in vitro and in vivo. This approach of using a silk fibroin dressing containing quercetin and functional motifs for wound healing has not been reported before, and it is the main focus of this study.

## 2. Materials and Methods

### 2.1. Materials

Unless otherwise specified, all chemicals, reagents, solvents, and TLC sheets were obtained from commercial sources (Tokyo chemical industry Tokyo, Japan, Sigma-Aldrich St. Louis, MO, USA, Spectrochem, Mumbai, India, and Avra Hyderabad, India). Cocoons of Bombyx mori were sourced from the Department of Sericulture in Tamil Nadu, India. Buffered paraformaldehyde (4%), PBS buffer, and lysozyme were purchased from Sigma-Aldrich. The NIH 3T3 fibroblast cell lines were acquired from the National Centre for Cell Science, Pune, India. All additional chemicals used in this research were chosen from the highest grade commercially available. The Gram-positive *Staphylococcus aureus* (ATCC 9144), *Enterococcus faecalis* (ATCC 35550), and Gram-negative *Pseudomonas aeruginosa* and *Escherichia coli* (ATCC 9027 and ATCC 25922) were obtained from the Institute of Microbial Technology, Sector 39A, Chandigarh, India, 160036. The male Wistar rats weighing 300–350 g for approximately 6–7 weeks were obtained from Venkateshwara breeders and laboratory animals in Bangalore, India.

### 2.2. General Procedure for the Synthesis of Quercetin Pyridyl Hydrazone 3a (QFM):

The QFM synthesis and characterization details are mentioned in the Supplementary Materials.

### 2.3. Silk Fibroin Extraction from Bombyx mori

The procedure for extracting pure silk fibroin (SF) from *Bombyx mori* cocoon was conducted following a highly efficient methodology, as detailed in our previously published report [27]. Firstly, the pupae were removed from the cocoon, and then the cocoon was subjected to a series of steps to eliminate sericin. It was washed, chopped, and simmered for 30 min in a 0.02 M sodium carbonate solution. After thorough cleaning with ultrapure water, the newly formed silk fibroin thread was left to dry overnight. A white SF thread weighing 25 g was subsequently placed into a solution containing lithium bromide with a concentration of 19.5 M, then heated at 60 °C for 8 h. The silk fibroin solution was subsequently dialyzed using Milli-Q water. Periodic replacement of the aqueous medium was carried out every four hours until a homogeneous solution of SF was achieved. Subsequently, the solution underwent freeze-drying to obtain pure SF powder, which was then kept at a temperature of 4 °C for subsequent use.

### 2.4. Fabrication of SF and SF-QFM Nanofibers Mat

Spun SF-QFM fibrous scaffolds were fabricated using an HO-NFES-040 electrospinning setup Holmarc’s, Kochi, India, following procedures outlined in our previous report [27]. In brief, an 8% (*w*/*v*) SF sponge and a 50 mg QFM drug homogeneous solution were prepared by dissolving in an HCOOH/CH_3_COCH_3_ (4:1) ratio. After a 5 h SF-QFM dissolving reaction in the presence of formic acid and acetone, the resulting homogeneous solution was electrospun using a 10 mL plastic syringe with a 24 G needle, applying 20 kV with a 1 mL/h flow rate. The spun collector was positioned 10 cm away from the needle tip. Both pure SF- and SF-QFM-spun fiber scaffolds underwent solvent evaporation before physiochemical and biological characterization. The resulting SF and SF-QFM fiber mats were initially washed with 80% *v*/*v* ethanol and then washed gently with distilled water at room temperature. To neutralize the spinning scaffolds, SF and SF-QFM mats were immersed in aqueous Na_2_CO_3_ at various concentrations and washed with distilled water, allowing them to remain at room temperature for 3 h. Finally, the SF and SF-QFM mats were vacuum-dried for up to 24 h and stored for further use [28].

### 2.5. NMR, FTIR, ESI-MS Measurement

The QFM characterization details are mentioned in the Supplementary Materials.

### 2.6. Thermal Stability Analysis

The thermal deformation of the bioactive QFM compound, SF, and SF-QFM nanofiber scaffold underwent examination by V4.4A TA Instrument, Eden Prairie, MN 55344, USA. The research included the determination of the melting point of a 10 mg sample of the QFM compound and the evaluation of the heat resistance properties of SF and SF-QFM scaffolds (measuring 5 mm) using a weight of 5 mg. The temperature range covered by the study was 0 to 800 °C, at a heating rate of 10 °C per minute [29].

### 2.7. Morphological Observation and Mechanical Property

The fiber structural characteristics and texture of formulated SF- and SF-QFM-spun fibers (10 × 10 mm^2^) were analyzed by FESEM. The SF and SF-QFM nanofiber scaffolds were subjected to gold sputter coating, and the surface morphology was examined with a 10 kV accelerating voltage. Using the ImageJ software version 1.54, 100 fibers were randomly selected, and their mean diameter was measured for the fabricated SF and SF-QFM scaffolds [30].

The dried rectangular prepared SF and SF-QFM nanofiber mats (10 × 50 mm^2^) were used to analyze the elongation ability and tensile strength using tensile test equipment (INSTRON Universal Testing Machine E-3000, Instron, Canton, OH, USA) at 0.2 mm/s speed [31].

### 2.8. Water Retention Capacity, Biodegradation Study, Contact Angle, and In Vitro Drug Release Study

The formulated SF and SF-QFM nanofiber scaffold (10 × 10 mm^2^) was immersed in pH 7.4 phosphate buffer saline (PBS) at room temperature for 72 h to determine its ability to retain water. Once the system had achieved equilibrium for swelling, samples of the swollen SF and SF-QFM matrices were collected at different time intervals. The mats’ surface moisture was eliminated using filter paper, hung for 1–2 min to eliminate any excess moisture, and subsequently weighed using a precision weighing balance; samples were tested in triplicates [32]. The absorption ratio was determined as

(1) $$\mathrm{Water}\;\mathrm{retention}\;(\%)=\frac{W1-W2}{W2}\times 100$$

*W*1 represents the sample’s original weight, and *W*2 reflects the sample’s weight following its absorption in the PBS solution.

The rate of SF and SF-QFM degradation was tested in vitro by soaking the fibrous mats (10 × 10 mm^2^) in PBS (7.4) containing 7–13 mg/L lysozyme solution and placed in a shaking incubator set at 37 °C. The degradation rate of the SF and SF-QFM nanofiber scaffolds was evaluated by measuring their biodegradability at different time intervals (2, 4, 6, 8, 10, 12, 14, and 16 days). Consistency was maintained, and for every time frame, a completely new enzyme solution was employed. The careful separation and washing of the spun mats were performed using double-distilled water after each interval; they were then allowed to dry in the air before being weighed, and samples were tested in triplicates. The determination of the biodegradation rate was accomplished by employing the subsequent equation [33].

(2) $$\mathrm{Degradation}\;\mathrm{rate}\;(\%)=\frac{Wi-Wf}{Wf}\times 100$$

The sample weight before degradation is indicated by *Wi*, while the sample weight after degradation is displayed by *Wf*.

The hydrophilicity and surface wettability of the fabricated SF and SF-QFM nanofibrous matrix were assessed via water contact angle using a KRUSS goniometer DSA25 analyzer, Hamburg, Germany. The SF and SF-QFM nanofiber mats were cut into squares (3 × 3 cm) and placed on the analyzer plate. The deionized water droplets were applied on the surface of the mats. Using the equipment software, the contact angles of the mats were examined and subsequently recorded [34].

The release of the therapeutic agent QFM from the SF nanofiber scaffold in pH 7.4 phosphate buffer saline was studied. Standard solutions with known amounts of QFM were prepared, and absorbance at 290 nm was used to create standard graphs. A 5 mg weight of fabricated spun SF and SF-QFM scaffolds in a square form was used to incubate in 5 mL of fresh phosphate-buffered saline at a temperature of 37 °C with gentle agitation for 72 h. At scheduled intervals, one milliliter of the acquired media was replaced with a fresh medium. The amount of QFM released was determined using a Jasco V-730 UV-vis spectrophotometer, Mary’s Court Easton, MD 21601 USA at 290 nm by comparing it with the standard plot [35].

### 2.9. Antimicrobial Activity, Antioxidant Assay, and In Vitro Cytocompatibility

The antimicrobial efficacy of the SF and SF-QFM nanofibrous dressings was assessed using the disk diffusion method against four bacterial strains: *Escherichia coli*, *Pseudomonas aeruginosa*, *Staphylococcus aureus*, and *Enterococcus faecalis*. Bacterial cultures were cultured on Nutrient agar via the streak plate technique to obtain distinct colonies. After 48 h of growth, individual colonies were selected and inoculated into the nutrient broth. The growth of the cultures was monitored using a UV-1800 spectrophotometer, Shimadzu, Dusseldorf, Germany at a wavelength of 600 nm, yielding a final OD of 1.0 after 24 h. For the disk diffusion method, Mueller–Hinton agar plates were utilized. A 100 μL inoculum was added to the agar plate and evenly spread across the entire surface using an L-rod. Antimicrobial-impregnated disks were carefully placed onto the agar surface using forceps. Following the placement of all disks, the plates were inverted, covered, and incubated in a 35 °C air incubator for 16 to 18 h. Triplicates were prepared for each sample. After the incubation period, the zone sizes were measured to the nearest millimeter using a ruler to determine the antimicrobial activity of the dressings [36].

The bioactive potential of QFM, resulting from its antioxidant activity, was evaluated using the DPPH test. DPPH (0.1 mM), along with 100 μL of CH_3_OH solution, was mixed at various concentrations of QFM (500, 300, 250, 100, 50, and 10 μg/mL). The mixture was then shaken and placed in a dark environment for up to 30 min. Subsequently, the DPPH quenching efficacy of the QFM-DPPH reaction mixture was examined at 517 nm and compared with the control. The inhibition percentage and the calculated IC_50_ value were determined, and ascorbic acid was used as the reference compound [37]. The DPPH radical neutralization capacity is estimated using this formula.

(3) $$\mathrm{DPPH}\%\;\mathrm{inhibition}=\frac{\left(absorbance\;of\;control-absorbance\;of\;the\;reaction\;mixture\right)}{absorbance\;of\;control}\times 100$$

The cytotoxicity of the QFM compound was evaluated in vitro using NIH 3T3 cells through the MTT assay. To begin, the NIH 3T3 cells were harvested and combined in a 15-mL tube after trypsinization. Subsequently, the cells were plated at a density of 1 × 10^5^ cells/mL cells/well (200 µL) in a 96-well tissue culture plate containing DMEM medium supplemented with 10% FBS and 1% antibiotic solution. The plate was then incubated at 37 °C for 24 h. Following this, the wells were rinsed with sterile PBS and treated with varying concentrations of the QFM compound in a serum-free DMEM medium. Each sample was replicated three times, and the cells were further incubated in a humidified 5% CO_2_ incubator at 37 °C for 24 h. After incubation, MTT solution (10 µL of 5 mg/mL) was added to each well, and the cells were incubated for an additional 2–4 h until purple precipitates became visible under an inverted microscope. To conclude the assay, the medium containing MTT was aspirated from the wells (220 µL) and washed with 1X PBS (200 µL). Finally, 100 µL of DMSO was added to dissolve the formazan crystals, and the plate was shaken for 5 min. The absorbance of each well was measured at 570 nm using a microplate reader (Thermo Fisher Scientific, Waltham, MA, USA), and the percentage cell viability was carried out using the formula below.

(4) $$Cell\;viability\;\%=\frac{Test\;OD}{Control\;OD}\times 100$$

### 2.10. Invitro Biocompatibility, Cell Adhesion, and Proliferation Studies

NIH3T3 fibroblast cells were cultured on both SF and SF-QFM nanofibers scaffolds. Immunocytochemistry was conducted at various time intervals (2, 4, and 6 days) to assess the extent of cell adhesion on the scaffolds. The procedure involved washing the cell-seeded scaffolds twice with DPBSA and fixing them with 4% paraformaldehyde (PFA) for 15 min. After gentle washing with DPBSA to remove any PFA residue, the scaffolds were permeabilized with 0.25% Triton X-100 for 15 min, followed by additional gentle washing with DPBSA. To visualize the cytoskeleton, the cells were stained with Phalloidin for 10 min and then washed twice with DPBSA. Subsequently, the cells were stained with Hoechst dye for 10 min to visualize the cell nuclei on the scaffolds. The scaffolds were gently washed twice with DPBSA and examined using a Fluorescent microscope [38]. The cell count from different images at different locations for each group (SF and SF-QFM) at 2–6 days was analyzed using ImageJ software version 1.54. The number of nuclei was quantified, and the average count was compared between the SF and SF-QFM groups at different time points.

### 2.11. Scratch Assay

The assay aimed to evaluate the efficacy of formulated SF and SF-QFM fibrous architecture for in vitro cell migration and wound healing. The migration ability of NIH 3T3 fibroblasts was assessed using the scratch assay in an in vitro setting over 24 h. The investigation sought to understand the ability of the fabricated scaffolds to promote cell migration, which is a crucial process in tissue regeneration and wound healing. The NIH 3T3 cells were grown and cultured using a 12-well culture plate with 2.5 × 10^4^ cells/well. Once the cells formed a complete layer, the cell monolayer was gently scraped with sterile 200 μL pipette tips. The sterile SF and SF-QFM bioactive extract at an equal level of concentration were applied to the culture plate and allowed to incubate following three PBS rinses. The effectiveness of in vitro wound healing was measured utilizing ImageJ analysis software version 1.54 [39,40].

### 2.12. In Vivo Wound Healing Study and Histological Analysis

The biomedical potential of therapeutically active QFM-loaded SF (SF-QFM) and native SF-spun fiber mats was tested in an in vivo preclinical wound healing experimental model using 300–350 g male Wistar rats that were 6–7 weeks old. The animal experiment procedures were approved by the Institutional Animal Ethical Committee (SRM College of Pharmacy, SRMIST, Kattankulathur, Chennai, India, IAEC No. 253/2021). The preclinical model was followed according to our previous study [27]. In brief, three randomly allocated groups of Wistar rats were employed in this in vivo model: an SF-QFM treated group (spun fibers nature; *n* = 6), a pure SF group (spun fibers nature; *n* = 6), and a control group (wrapping with cotton gauze; *n* = 6). Each rat’s back was shaved after proper anesthesia, and a full-thickness skin wound with a diameter of 10 mm was created [41,42]. Constructed spun implants were delicately positioned over the injured area as promptly as possible, ensuring the well-maintenance and periodic observation of all test animals at 3-, 6-, and 9-day intervals. After each interval (3, 6, and 9 days), rats in each group were euthanized, and the healed tissues were collected and assessed to measure the effectiveness of the group’s recovery. The wound region was examined through camera photographs to determine the wound recovery rate. The wound closure percentage in rats was calculated using the following equation:

(5) $$\mathrm{Wound}\;\mathrm{area}\;(\%)=\frac{A1-A2}{A1}\times 100$$

The initial size of the wound area, measured on the day of the surgery, is indicated by *A*1. The wound area measurements after 3, 6, and 9 days following the original injury are denoted by *A*2.

The wound site tissues obtained on postoperative days 4 and 8 were embedded with paraffin after being stored in 10% formalin. Sections with a thickness of 4 μm were subjected to hemotoxylin–eosin (H&E) and Masson’s trichrome stains [43,44]. Histological staining using hematoxylin and eosin (H&E) was carried out to examine the presence of inflammatory cells, the progress of re-epithelialization, the formation of new fibroblast cells, and the production of microvasculature and granulation tissue in the wounded area at both the 4-day and 8-day time intervals. The Masson trichrome stain was used to detect the presence of newly formed collagen in the wound. The histological sections were examined under a Zeiss Axioscope microscope, Wetzlar, Germany [45].

### 2.13. Statistical Analysis

All tests were conducted with three replicates (*n* = 3). Statistical comparisons were performed using GraphPad Prism (5.0) with two-way ANOVA, and statistically significant values were denoted as ** *p* < 0.01 and *** *p* < 0.001.

## 3. Results

Recent progress has highlighted the capability of nano bioengineering methods to enhance the therapeutic attributes within the combination of biopolymer systems for the discovery of regenerative medicine. Employing this approach is crucial in transforming bioactive compounds from their larger state to nano-sized particles, particularly vital in tissue engineering and biomedical applications. We developed and evaluated the effectiveness of a new electrospun silk fibroin nanofibrous mat infused with bioactive quercetin containing morpholine–pyridine motifs (QFM) for promoting wound healing in a rat model.

### 3.1. Chemistry

**Scheme 1:**
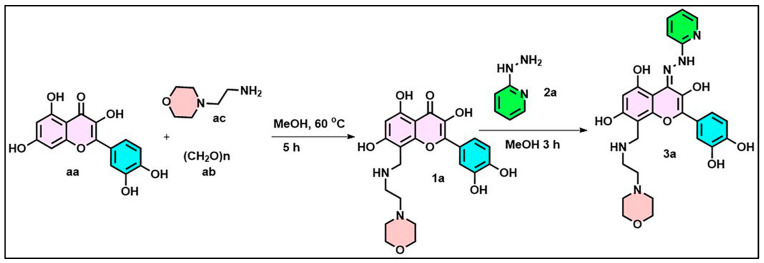
Schematic illustration for the synthesis of quercetin pyridyl hydrazone (QFM).

Various spectroscopy techniques, including ESI-MS, FT-IR, ^1^H NMR, and ^13^C NMR, confirmed the structure of the synthesized compound QFM (Scheme 1). In ^1^H-NMR of 3a (QFM), at a downfield chemical shift of 10.35 ppm, a proton with an alcohol functional group (α-OH proton) exhibits broad and high lability. In addition, at 9.55 ppm, four protons in the phenolic group (Ph-(OH)_4_) show extensive and high lability. In addition, at 9.50 ppm, a single proton in the NH group displays comprehensive and high lability. Aromatic protons were observed within the chemical shift range of 8.46 ppm to 7.49 ppm, with eight protons identified. The characteristic benzylic protons (CH_2_) exhibited a chemical shift at 4.32 ppm, followed by the appearance of the (4H) or ethene (CH_2_)_2_ proton (N-CH_2_-CH_2_-N) and eight aliphatic protons of morpholine (O-(CH_2_-CH_2_)_2_-N) in the chemical shift range of 2.12 to 0.9 ppm (Figure S1). In the context of ^13^C NMR spectroscopy, the a-hydroxy–imine compound tautomerizes to the keto–enol form. Thus, the carbon atom in issue demonstrates a chemical shift of approximately 200.01 ppm. In addition, the aromatic carbon atoms associated with this compound are detected between 157.45 and 113.01 ppm. A distinct set of aliphatic carbon atoms appears between 62.57 and 31.98 ppm, providing definitive evidence for the precise structure of compound **3a** (as depicted in Figure S2). The ESI-MS technique validates the precise structures and molecular masses of compound **3a**. Chemical formula: C_27_H_29_N_5_O_7_; calculated mass ESI-MS (*m*/*z*): 535.21. Chemical formula: C_27_H_29_N_5_O_7_Na; calculated mass ESI-MS (*m*/*z*): 558.30; found mass ESI-MS (*m*/*z*): 558.30 [M + Na]^+^ (Figure S3).

### 3.2. FTIR Analysis

**Figure 1:**
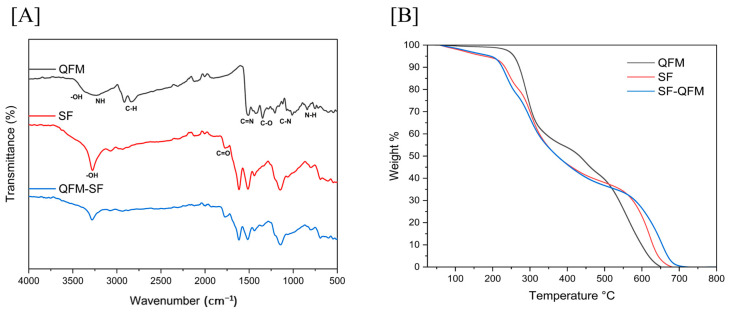
(**A**) ATR-FTIR spectrum of synthesized QFM, SF, and SF-QFM fibrous scaffolds; (**B**) thermogravimetric analysis (TGA) of QFM, SF, and SF-QFM scaffolds.

The functional group of QFM, SF, and SF-QFM nanofibers scaffold was confirmed by attenuated total reflectance–FTIR spectral analysis (Figure 1A). The stretching frequency of the C=N functional group in the flavanone imine moiety of quercetin pyridyl hydrazone (**3a**) causes the spectrum at 1629 cm^–1^ in QFM. Secondary amines of pyridyl hydrazone (**3a**) (C=N-NH) have a weak absorption band in the 3300–3000 cm^−1^ region, corresponding to N–H stretching vibrations. These bands are less intense and more distinct than the O–H segments in compounds with one N–H bond. An extra amine band is observed at 910–665 cm^−1^, and N–H undulating motion, exclusively in secondary amines, gives a solid and broad spectral band. Amines’ C–N stretching vibrations often appear as medium bands at 1250–1020 cm^−1^. Aromatic amines have a strong 1335–1250 cm^−1^ vibrational band. The phenolic O–H weak stretching mode of (**3a**) has a weak, narrow absorption band at 3482 cm^−1^ in FTIR spectroscopy. In the QFM (flavanone α-OH) example, the absorption band 3290 cm^−1^ absorption peak characterizes the polymeric hydrogen bond. While C–N stretching is 1143 cm^−1^, the N–H-wag secondary amine may cause this shift. The QFM stretching bands of the C–H groups exhibit two maxima at 2943 cm^−1^ and 2880 cm^−1^, whereas the C–O–C functional groups’ asymmetric and symmetric stretching vibrations are at 1294 cm^−1^ and 1184 cm^−1^. The 1243 cm^−1^ peak indicates C–H bond vibrational modes, whereas the 1045 cm^−1^ peak shows C–O and C–H bond stretching and bending. The above-described peaks depicted confirmation of the expected structure of the SF-QFM fibrous scaffold in Figure 1A.

### 3.3. Thermal Stability

The thermal analysis of electrospun nanofibers blended with additional compounds provides valuable insights into their degradation and transformation. It is essential to assess the physical and thermal properties of biopolymers like SF and hybrid biopolymers based on bioingredients to understand their thermal resistance, chemical structure, and potential applicability in tissue engineering. TGA was conducted to assess the thermal behavior of the QFM, SF fibrous mat, and SF-QFM fibrous mat. Incorporation of the QFM molecule into the SF fibrous mat did not result in any adverse effects or significant differences in stability. The SF-QFM material’s TGA curve indicates moisture’s evaporation between 50 and 70 °C. In contrast, the material’s disintegration occurs in the 290 to 300 °C range. The synthesized QFM and prepared SF nanofibers displayed melting temperatures ranging from 230 to 240 °C, with denaturation peaks observed between 285 and 290 °C (Figure 1B). The strong binding ability of the integrated QFM within the SF fibrous structure indicates that there are no significant changes in thermal stability or undesired modifications after incorporation. Figure 1B illustrates the macromolecular structure of an interconnected biopolymeric network (SF-QFM) as thermally stable, showing a steady rate of disintegration after QFM impregnation. These results are promising, as thermally stable nanomaterials are known to preserve the structural properties necessary for distinct biological attributes in various biomedical applications.

### 3.4. Morphological Observation and Mechanical Properties

**Figure 2:**
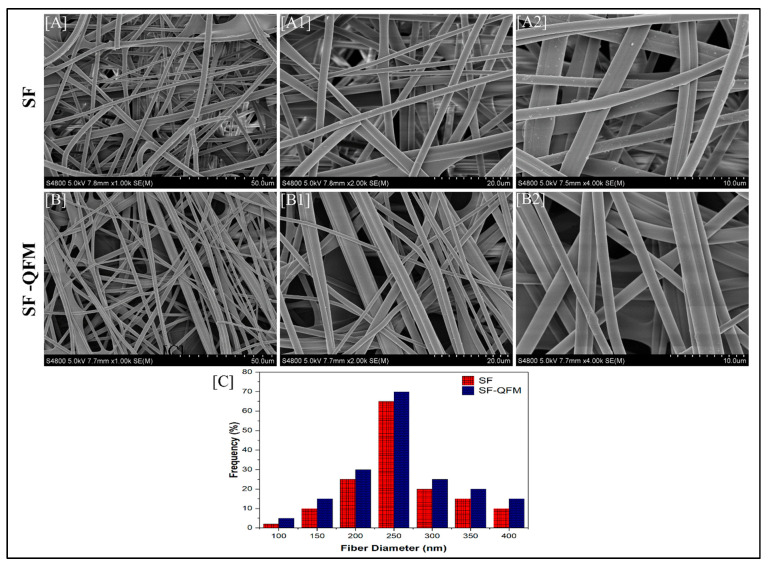
(**A**–**A2**) SEM micrographs of the SF nanofibers; (**B**–**B2**) SF-QFM nanofibers; (**C**) fiber diameter distribution graph of the SF and SF-QFM electrospun nanofibers.

FESEM was used to examine the structure, morphology, diameter, and compactness of SF-QFM and SF nanofibers. The SEM pictures of SF-QFM and SF electrospun fibrous scaffold illustrate a fibrous architecture that is uniform, bead-free, smooth, and randomly oriented, as shown in Figure 2A–B2. The high surface area-to-volume ratio of nanofibers offers numerous advantages in wound healing, including enhanced cell adhesion, proliferation, enhanced oxygen penetration, and improved drug loading and release capabilities. The fiber diameters and distribution accuracy of both SF and SF-QFM scaffolds were determined by randomly selecting 100 fibers from each scaffold and analyzing them using ImageJ software. The addition of QFM to the SF-QFM nanofiber mat did not produce any significant changes in the formation of a uniform fibrous structure. Figure 2C clearly shows that the average diameters of the SF and SF-QFM nanofiber mats are about 217 nm and 223 nm, respectively. The SEM images of fibrous mats with and without the presence of QFM molecules in the fibrous architecture revealed the fibers’ exceptional smoothness, uniformity, and compactness. In addition, the SF-QFM scaffold incorporates the potent biologically active molecule QFM, making it a highly effective tool for combating bacterial infections. The distinctive characteristic of the SF-QFM-spun architecture is its tremendous ability to amplify the therapeutic impact against infections and stimulate cellular activities. Moreover, the sustained release of drugs facilitated by this spun architecture can significantly contribute to tissue reconstruction by stimulating cell proliferation and differentiation in targeted tissues. The study’s findings validate the efficacy of the SF-QFM scaffold, which possesses potent multifunctional properties, in promoting healing in infected and injured tissue regions. Furthermore, the surface morphology of the scaffold is convenient, while its porous architecture helps maintain essential physicochemical properties, further accelerating the healing process. Consequently, strong antibacterial and antioxidant moiety-loaded SF-QFM-spun morphology and their bioactive characteristics surely contribute to prolonged drug administration, making them highly suitable in a therapeutic setting for encouraging tissue regeneration and fending off bacterial infections.

The inherent mechanical attribute of nanofiber scaffolds is a key determinant that impacts the appropriate regenerative therapeutics formulation for reinstatement functionality in impaired or wounded tissues. The mechanical properties of the SF-QFM and SF nanofiber matrix were evaluated by examining key mechanical parameters, namely tensile strength and rupture elongation at the point of failure in the nanofiber mat. When comparing the SF-QFM matrix to the pure SF matrix, it was observed that the SF-QFM matrix exhibited higher mean elongation at break and greater tensile strength. The results obtained from Table 1 indicate that the mean elongation at break for the SF-QFM matrix was approximately 10.38 ± 0.7 MPa, whereas for the pure SF matrix, it was 9.47 ± 0.3 MPa. Similarly, the tensile strength of the SF-QFM matrix was approximately 5.07 ± 1.1%, while for the pure SF matrix, it was 3.75 ± 1.2%. The SF-QFM nanofibers have the ability to generate significant intermolecular forces within their structure. Therefore, quercetin containing morpholine and pyridine functional motifs might significantly increase affinity within the fibers’ structure. It may facilitate accurate and efficient elasticity to envelop the entirety of the damaged tissues. As per our research, it has been observed that the SF fibrous scaffold impregnated with QFM exhibited remarkable mechanical prowess, thereby enhancing the wound-healing process. Consequently, the findings denote that the SF-QFM scaffold possesses remarkable biomimetic surface morphology owing to its high thermal stability, sustained permeability, controlled degradability, and exceptional flexibility. These attributes are conducive to enhancing functionalities such as biocompatibility, superior cell adhesion, and cell viability, thereby facilitating the acceleration of the healing process.

### 3.5. Water Retention Capacity, Biodegradation Study, Contact Angle, and In Vitro Drug Release Study

**Figure 3:**
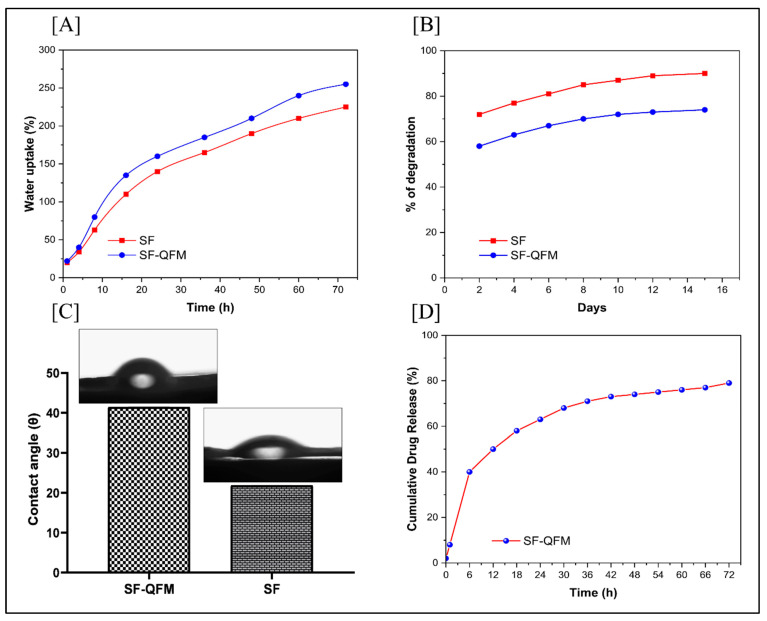
(**A**) In vitro water retention ability; (**B**) degradation profiles; (**C**) water contact angle; (**D**) cumulative drug release profiles of SF and SF-QFM nanofibers matrix.

The prepared spun material was analyzed for its water uptake capacity and equilibrium swelling behavior using PBS buffer with a pH of 7.4. Figure 3A shows the water absorption capabilities of the SF-QFM and SF nanofiber dressings. The QFM-impregnated silk fibroin nanofiber dressings exhibited a greater capacity to bind water than the SF nanofiber dressings, owing to their high force of bonding interconnection and QFM water-attracting property. Incorporating the QFM into the SF nanofibers increased the swelling rate by approximately 160% and 210% after 24 and 48 h of soaking in PBS buffer, respectively. Furthermore, the SF fibrous mat without QFM demonstrated moderate swelling with a water uptake percentage of approximately 140% at 24 h and 190% at 48 h. After 72 h, it was discovered that the entwined smooth SF-QFM and SF fibrous mats had water uptake percentages of approximately 255% and 225%, respectively. The SF-QFM fibrous system showed improved water uptake ability compared to the SF fibrous system. The SF-QFM fibers have strong interactions between SF and QFM, which improve affinity, allow for quick nutrient absorption, and enhance biocompatibility and infiltration efficiency in the nanofibrous platform. The SF-QFM nanofibrous scaffold can absorb water, thus increasing oxygen levels in damaged tissue, facilitating nutrient delivery, and aiding in the removal of residual waste, thereby promoting faster wound healing.

Developing a biomaterial that possesses sustained release and controlled biodegradation capabilities incorporating SF-based fibrous mats can significantly enhance the bioactivity of damaged tissues and, in turn, accelerate the process of new tissue regeneration and promote effective healing. The investigation of enzymatic degradation is crucial in establishing the correlation between the degradation rate and structure of fabricated fibrous materials and aids in comprehending the accessibility of enzymes. The research on regenerative medicines makes creating more interactive and practical biomaterials easier. The prepared SF-QFM and SF fibrous scaffold rate of enzymatic biodegradation was determined in vitro through immersion in a lysozyme solution (7–13 mg/L) for 15 days. As depicted in Figure 3B, the degradation rate was determined by analyzing the fibrous scaffolds’ weight loss at various time intervals. After 15 days, approximately 90% of silk fibroin fibrous scaffolds had degraded. The rapid degradation was caused by the silk fibroin’s entirely hydrophilic nature. After 15 days, the degradation rate for SF-QFM fibrous material was 74%. The sustained regulation of the QFM-containing SF nanofiber’s absorption characteristics during the water uptake process is the main factor influencing the efficient progressive enzymatic breakdown over SF-QFM. The SF-QFM nanofibrous scaffold shows a moderate hydrophilic nature and favorable flexibility, facilitating the interaction between QFM and SF, and the formation of intermolecular covalent bonds results in a high affinity and enhanced durability of the scaffold. Thus, the degradation rate was regulated by QFM in conjunction with SF to serve as an innovative bioactive material for wound healing in the presence of controlled decomposition.

Compared to the SF-QFM fibrous scaffold, the SF fibrous scaffold has good hydrophilic properties (Figure 3C). The water contact angle was about 21.8° at 10 s, and the SF-QFM was nearly 41.4° at 10 s due to the incorporation of QFM hydrophobic properties. However, the SF-QFM nanofiber can instantly infiltrate water, which results in noticeable variations in surface wettability. Utilizing the SF mat infused with bioactive QFM can enhance cellular integration cell viability and facilitate the development of new tissue by effectively eradicating harmful bacterial infections. According to the study, the QFM with SF electrospun mat architecture demonstrated effective water absorption, which could readily encourage the improvement of functionalities like improved cell adhesion, increased cell viability, and biocompatibility, facilitating the acceleration of the healing process.

Electrospun mats made of silk fibroin and QFM are intended to promote sustained medication release while promoting cell adhesion, proliferation, and migration to the wounded area. The cumulative QFM release profile from SF-QFM electrospun mats was performed with PBS (with a pH of 7.4) for up to 72 h, as shown in Figure 3D. The observed data indicate that QFM was rapidly released within the first 6 h, which may have been caused by QFM that had accumulated over the pores of fibrous mats. Therefore, the preliminary release was found to be approximately 40%. Utilizing homogeneous nanofibers, consisting of a combination of hydrophilic and hydrophobic structures of SF and QFM, exhibits robust binding strength and gradual biodegradation. As a result, the system facilitates controlled release throughout 12 to 72 h. Hence, the QFM-embedded SF fibrous system demonstrated a cumulative release of 79% within a 72 h release level, which is deemed suitable for effectively reducing bacterial load and prevalence at the site of an infected wound, thereby promoting expedited healing.

### 3.6. Antibacterial Activity, Antioxidant Assay, and In Vitro Cytocompatibility

**Figure 4:**
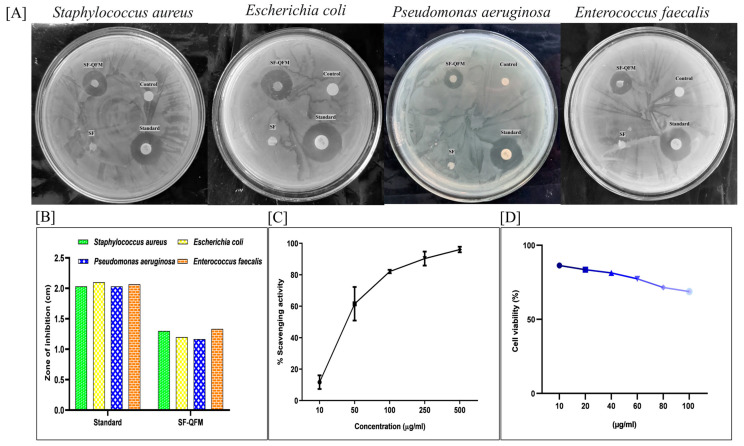
(**A**) Antimicrobial activity of control, standard, and SF and SF-QFM nanofibers scaffolds samples against *S. aureus*, *E. coli*, *P. aeruginosa,* and *E. faecalis*. (**B**) ZOI difference in SF-QFM and standard. (**C**) DPPH free radical scavenging activity percentage against various concentrations of QFM. (**D**) Results of the in vitro cytotoxicity (MTT) assay for synthesized QFM.

The SF-QFM nanofibrous scaffold exhibited significant antimicrobial activity, especially against *S. aureus* and *P. aeruginosa*. These two types of bacteria frequently cause wound bed infections. The disc diffusion method evaluated the antibacterial properties of SF, SF-QFM nanofiber membranes against *E. coli*, *P. aeruginosa*, *S. aureus,* and *E. faecalis*. The QFM demonstrated positive activity against both Gram-positive and Gram-negative bacteria (Figure 4A). The results indicate that *S. aureus*, *E. faecalis*, *P. aeruginosa*, and *E. coli* exhibit growth near both SF nanofibers and blank filter paper (negative control group), implying the absence of antibacterial activity in SF nanofiber membranes. The antibacterial zone diameter against *S. aureus* (*E. faecalis*) and *E. coli* (*P. aeruginosa*) was 1.30 mm (1.33 mm) and 1.20 mm (1.17 mm), respectively, for the SF-QFM nanofiber membrane (Figure 4B). The SF-QFM nanofiber membrane exhibited good antibacterial activity compared to the positive control group. The nanofibrous scaffold material shows promise as a potential biomaterial for preventing infection near a wound. SF-QFM nanofibrous drug-eluting dressings can release antibiotics gradually or in response to specific triggers, enhancing treatment effectiveness. Additionally, local delivery reduces healthcare costs by minimizing the need for prolonged systemic therapy or hospital stays and offers a novel and cost-effective way to enhance wound healing.

**Figure 5:**
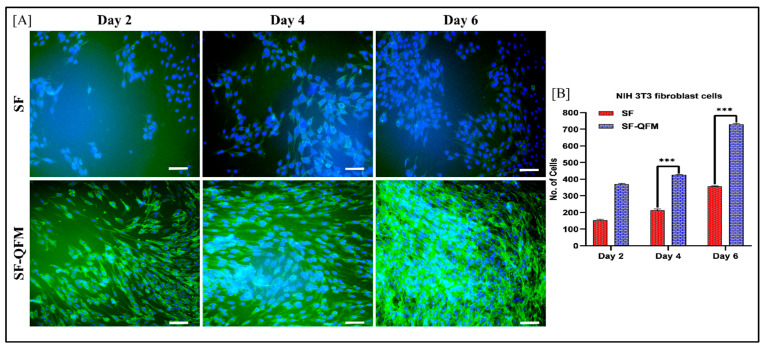
(**A**) In vitro biocompatibility of SF and SF-QFM nanofibers scaffolds. (**B**) Quantification of NIH 3T3 fibroblast cells at various time points (scale bar: 100 μm). *** *p* < 0.001.

During the inflammatory phase of wound healing, immune cells generate ROS to eliminate bacteria and debris. While this is a necessary part of the healing process, excessive ROS levels can lead to tissue damage and delay wound healing. The compound QFM exhibited high antioxidant activity, as demonstrated by its ability to scavenge DPPH (Figure 4C). The IC_50_ value of 43.61 μg/mL indicates that QFM acts as scavengers to counteract the accumulation of ROS and protect cells from oxidative damage by stabilizing free radicals through electron donation and activating antioxidant enzymes that detoxify ROS by reducing oxidative stress to create a favorable environment for the healing of wounds. The antioxidant activity of QFM was further confirmed through cytotoxicity assays and cell growth analysis. SF-QFM significantly supported cell growth compared to the control, as evidenced by the increased cell density observed in cell imaging (Figure 5A) at the 6-day mark.

The optimization of cell viability was observed in both the newly synthesized QFM and native quercetin. Figure 4D displays the results, showing that cells thrived on QFM at all dilutions, indicating its cytocompatibility and non-toxic nature. QFM cell viability consistently remained above 70% at all dilutions (Figure 4D), while native quercetin only showed less than 55% viability at all dilutions (Figure S4), confirming QFM’s advantageous properties for NIH 3T3 cell viability. The novel QFM bioactive agent exhibited enhanced viability of NIH 3T3 cells across all concentrations, demonstrating its superior efficacy over native quercetin. Therefore, we have decided to utilize the synthesized QFM incorporated into SF (SF-QFM) for further studies to enhance wound healing activity in preclinical settings. Additionally, the presence of the biologically active moiety in QFM enables it to maintain good cell viability in NIH 3T3 cells even at high concentrations after 24 h of incubation. This further confirms that QFM is a better option for various desired concentrations and formulations to enhance cell proliferation and growth.

### 3.7. In Vitro Biocompatibility, Cell Adhesion, and Proliferation Studies

The results from Figure 5A,B demonstrated that the SF-QFM scaffold exhibited a significant enhancement in cell adhesion and proliferation, indicating its non-toxic nature, high biocompatibility, and ability to promote cell–cell interactions within the NIH 3T3 cells throughout the entire experiment. The incorporation of QFM in the SF-QFM mats further improved their bioactive properties, facilitating accelerated cell proliferation, migration, and growth, consequently enhancing extracellular matrix functions and tissue regeneration. Comparatively, the SF-QFM group showcased a significantly increased fibroblast cell proliferation without any adverse effects when compared to the SF group, with a noticeable difference observed in NIH 3T3 fibroblast cells from day 2 to day 6 of incubation. This finding was supported by the average nucleus count analysis, confirming the biocompatibility and cell proliferation of both SF and SF-QFM dressings. On the other hand, the pure SF mat exhibited a decrease in fibroblast production at all stages. The addition of QFM, known for its multifunctional properties, degradation resistance, mechanical strength, and thermal stability, had a synergistic effect with silk fibroin protein, significantly enhancing cell–cell integration and proliferation. The SF-QFM mat showed distinct results compared to the pure SF mat after 4 and 6 days of incubation. The SF-QFM mat, due to its exceptional cell permeability and incorporation of bioactive agents, promoted the accelerated synthesis, adhesion, and proliferation of fibroblast cells, which underscores the mat’s adaptability, biocompatibility, and accessibility in the realm of tissue engineering. Moreover, the SF-QFM fiber scaffold promoted enhanced cell adhesion and NIH 3T3 fibroblast cell proliferation, thereby facilitating wound healing within a short duration.

### 3.8. Scratch Assay

**Figure 6:**
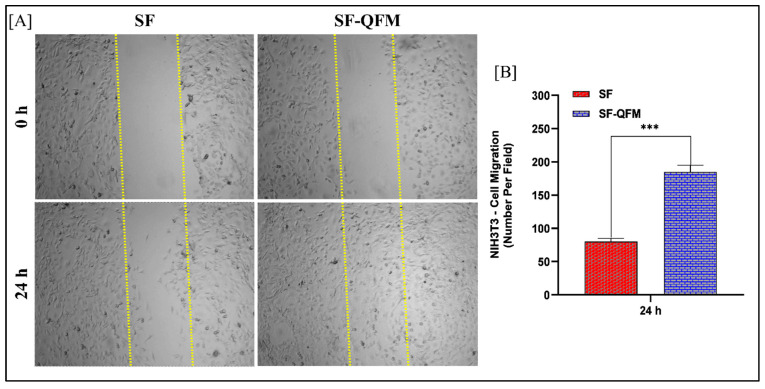
(**A**) In vitro wound healing (scratch assay) study of SF and SF-QFM fiber scaffolds. (**B**) Quantification of NIH 3T3 fibroblast cells at various time points (scale bar: 100 μm). *** *p* < 0.001.

The results showed a significant increase in cell migration within the scratched region of NIH 3T3 cells when cultured on QFM-loaded silk fibroin fibrous dressings Figure 6A. Enhanced migration can be attributed to the accelerated cell–cell interaction facilitated by incorporating QFM into the silk fibroin fibrous dressings, compared to dressings made solely from native SF fibers. The findings indicate that even after a 24 h incubation period, cell migration showed a positive and anticipated response in the SF-QFM fibrous dressings (Figure 6B).

### 3.9. In-Vivo Determination of the Wound Healing Activity

**Figure 7:**
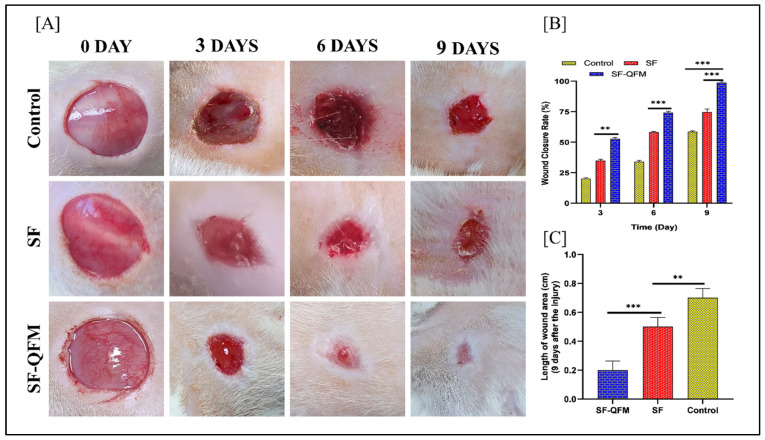
(**A**) Representative photographs depict the progression of wound healing; (**B**) the wound closure rates; (**C**) quantification of length of wound area at day 9. The significant difference (** *p* < 0.01, *** *p* < 0. 001).

The objective of this study was to assess the effectiveness of the control group, as well as the SF and SF-QFM mats, in promoting wound healing in a rat model within a timeframe of 3 to 9 days. The wound closure effectiveness rate of the treatment groups, SF and SF-QFM, was compared to that of the control group in the research to assess their capacity for skin wound healing. Figure 7A illustrates that the group treated with SF-QFM exhibited faster wound healing compared to the groups treated with pure SF and the control group. The healing process was substantially improved by the QFM-embedded SF nanofibrous mat, which possesses multifunctional capabilities, as evidenced by the observed improvement from the initial to the final interval (3–9 days). Figure 7B shows that the wound closure rates of SF-QFM after 3–9 days were 53.66%, 74.33%, and 100%, respectively. The healing rate was determined by conducting a comparative analysis between the native SF group and the control group, specifically in terms of their ability to reduce wounds. Furthermore, in comparison to the pure SF group, the SF nanofibrous mat infused with QFM exhibited superior re-epithelialization and wound healing after 9 days. Figure 7C illustrates that even after 9 days of observation, the wound in the cotton gauze control group took longer to close due to its inadequate permeability and limited infiltration capacity. By measuring the length of the wound area after 9 days, the effectiveness of wound healing in the treated and untreated groups was evaluated. However, after nine days of monitoring, the treated group’s total capacity for healing was assessed. The SF-QFM group showed quicker recovery at the 6-day point than the other groups because it has high biocompatibility, versatile therapeutic characteristics, and increased cell migration capacity. Six days after the injury, there were noticeable changes between the SF-QFM and native SF group. For the SF-QFM, SF, and control groups, the measured length of healing wound regions was around 0.21 ± 0.02, 0.54 ± 0.02, and 0.76 ± 0.01 mm, respectively. As a result, the SF-QFM group had considerably more significant alterations from 6 to 9 days than the SF and control groups. Gross pictures of the treated and untreated wound sites in Figure 7B,C were examined to identify these variations. A full-thickness cutaneous lesion treated with the multifunctional SF-QFM nanofibrous mat heals more quickly by encouraging cell migration and proliferation, re-epithelialization, differentiation of epidermic cells, and the production of new granulation tissue. After six days, 74 ± 3% of the wounds in the SF-QFM-treated group had healed, compared to 58% and 34% in the pure SF and control groups, respectively, demonstrating a regression in the healing process. The results from both in vitro and in vivo tests demonstrate the excellent multifunctional properties of the QFM-loaded SF electrospun fibrous mat for expediting the healing of infected wounds.

### 3.10. Histological Examination

**Figure 8:**
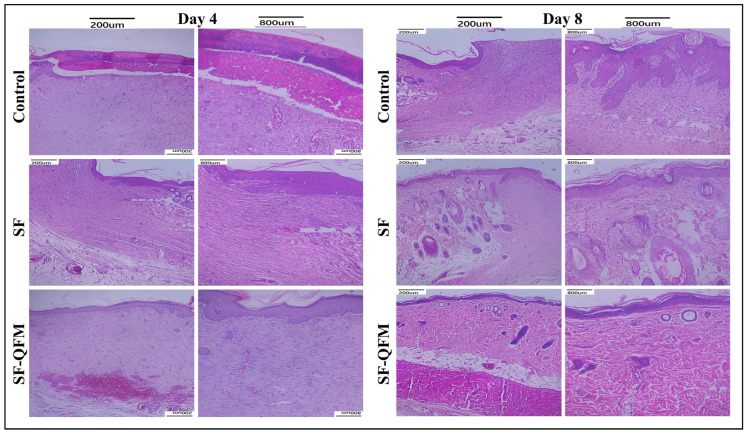
H&E staining image of the histological section of control, SF, and SF-QFM fibrous scaffolds on the fourth and eighth days (200× and 800×; scale bar 100 μm).

Our study observed varying degrees of inflammation infiltration in three distinct groups. The control group showed a significant increase in inflammatory cells, blood vessel injury, hemorrhage, and ulceration due to the limited permeability, biocompatibility, and therapeutic properties. In contrast, the treated SF-QFM group exhibited a minimal presence of inflammatory cells on the fourth day of examination when compared to the SF group, as depicted in Figure 8. On the fourth day after the surgery, noticeable variations were observed between the groups treated with SF-QFM and pure SF. Furthermore, the incorporation of morpholine and pyridine functional motifs containing quercetin (QFM) into the SF fibers, known as SF-QFM, can enhance hemostasis activation, thereby reducing the risk of excessive bleeding. Moreover, SF-QFM has the potential to support the growth and development of blood vessels by effectively preventing rupture and damage to them. The level of vascularization observed in the SF-QFM group was found to be significantly higher compared to both the SF fibrous group and the control group on the fourth day. The SF-QFM group demonstrated a significantly higher density of blood vessels compared to both the SF fibrous group and the control group. The findings suggest that the SF-QFM group had a positive impact on promoting vascularization during the early stages of wound healing. The results indicate that there was an interaction between the SF-QFM molecules occurring at an earlier stage, which facilitated the chemotactic process, leading to the migration of endothelial cells and the formation of capillary vessels in the wound area, which consequently accelerated the healing process within a shorter timeframe. Additionally, on the fourth day after the operation, we observed that the SF-QFM dressing exhibited well-organized granulation tissue and moderate epithelium compared to the other groups. It is worth noting that the SF-QFM dressing also showed a faster development of homogeneous mature granulation tissue, leading to accelerated epidermal regeneration. As a consequence, by day four, the healing rate was inadequate in both the SF and control groups, which failed to exhibit an improvement in granulation tissue regeneration function or early-stage epidermal production. As opposed to the SF and control groups, the SF-QFM group demonstrated organized neovascularization, progressive epithelialization, and enhanced fibroblast migration on day 8. On day eight, there was a lack of epithelial migration and granulation tissue formation in the control group, suggesting a decelerating rate of wound healing. Compared to the pure SF group, the SF-QFM-treated group exhibited well-stratified epithelium differentiation and freshly arranged homogeneous dense granulation tissue at an earlier stage. Furthermore, the SF-QFM group demonstrated superior growth in dermal papillae, capillary vessel production, basal cells, hair follicle formations, and sebaceous tissue within eight days after the treatment compared to the other groups. The SF-QFM fibrous mat group exhibited nearly total healing, accompanied by superior arrangement and remodeling of connective tissue, which resulted in a more regular structure. The favorable results observed can be attributed to the SF-QFM fibrous mat’s multifunctional properties that facilitate wound healing. Therefore, our findings suggest that the SF-QFM fibrous mat has immense ability as a wound dressing.

**Figure 9:**
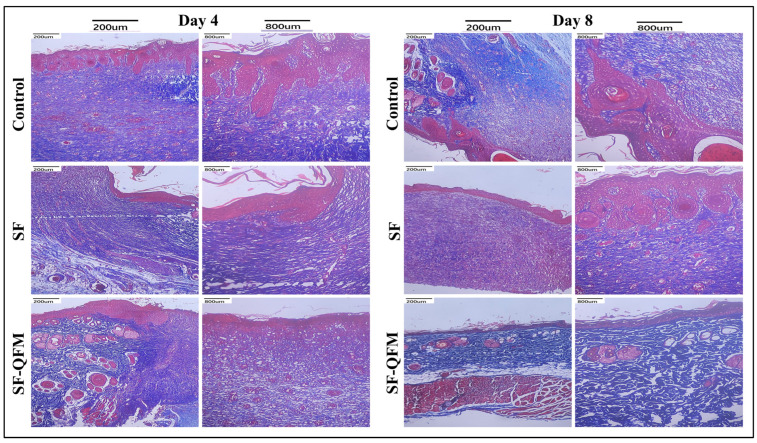
Masson’s trichrome staining images of control, SF, and SF-QFM fibrous scaffolds on the fourth and eighth days (200× and 800×; scale bar 100 μm).

As depicted in Figure 9, the SF-QFM mat displayed blue-stained collagen fibers on the fourth day of the experiment, which signified the initiation of tissue proliferation. At this time, the quantity of red-stained muscle fibers remains minimal. Comparatively, the other groups show less collagen fiber formation, and the control group has the highest number of muscle fibers, suggesting that it has yet to enter the tissue proliferation phase. By the eighth day, the SF-QFM group showed further increased collagen deposition, extending to the central area of the wounds with regular arrangements. Furthermore, this group exhibits a notable proliferation of newly formed capillaries and hair follicles, which exceeds the negligible quantity of blue-stained collagen fibers observed in the SF and control groups. These results indicate that the SF-QFM dressing promotes the best healing response, and the histomorphological evaluation strongly supports the capability of the SF-QFM dressing to enhance wound healing.

## 4. Conclusions

In this study, we successfully developed a multifunctional QFM bioactive molecule embedded within a silk fibroin-spun nanofiber scaffold for in vivo skin wound healing applications. Incorporating the QFM moiety into the SF-QFM mat resulted in a fibrous system with various therapeutic properties. The SF-QFM mat exhibited strong antibacterial properties, effectively preventing bacterial infections in the wounded area. Moreover, it served as a potent scavenger, neutralizing reactive oxygen species and safeguarding cells against oxidative damage. As a result, both in vivo and in vitro wound models showed higher effectiveness in promoting faster wound healing than the SF mat without QFM. Furthermore, this bioinspired spun SF-QFM nanofiber scaffold has exhibited exceptional mechanical and thermal stability, consistent effectiveness in drug release, and regulated degradability attributes that play a significant role in facilitating cell integration, migration, and proliferation, which are vital for the advancement of granulation tissue, re-epithelialization, and neovascularization processes. Our study’s biological and physicochemical results are encouraging that the SF-QFM, a fiber scaffold laden with QFM, exhibits robust antibacterial and antioxidant properties while maintaining excellent biocompatibility. It is particularly suitable for rapidly healing wounds in patients with elevated infection risk. Overall, the combination of an efficient therapeutic nanofiber mat holds the desirable ability as a multifunctional biomaterial to facilitate wound repair and regeneration, making it an attractive option for addressing the needs of patients with complex wounds.

## Figures and Tables

**Table 1:** Tensile properties of SF and SF-QFM nanofibrous scaffolds.

Sample	Mean Tensile Strength (Mpa)	Mean Extension at Maximum Load (mm)	Mean Elongation at Break (%)	Mean Elongation at Break (mm)
SF	9.47 ± 0.3	2.41 ± 0.6	3.75 ± 1.2	5.68 ± 2.8
SF-QFM	10.38 ± 0.7	3.64 ± 0.3	5.07 ± 1.1	6.32 ± 0.8

## Data Availability

The data used to support the findings of this study are available from the corresponding author upon request.

## Supplementary Materials

The following supporting information can be downloaded at: https://www.mdpi.com/article/10.3390/pharmaceutics16040462/s1, Figure S1: ^1^H NMR Spectrum of compound **3a**; Figure S2: ^13^C NMR Spectrum of compound **3a**; Figure S3: ESI-MS spectrum for compound **3a**; Figure S4: Results of the in vitro cytotoxicity (MTT) assay for Quercetin.

## References

[1] Byrd A.L., Belkaid Y., Segre J.A. (2018). The Human Skin Microbiome. Nat. Rev. Microbiol..

[2] Dandona R., Kumar G.A., Gururaj G., James S., Chakma J.K., Thakur J.S., Srivastava A., Kumaresh G., Glenn S.D., Gupta G. (2020). Mortality Due to Road Injuries in the States of India: The Global Burden of Disease Study 1990–2017. Lancet Public Health.

[3] Graves N., Phillips C.J., Harding K. (2022). A Narrative Review of the Epidemiology and Economics of Chronic Wounds. Br. J. Dermatol..

[4] Rezvani Ghomi E., Khalili S., Nouri Khorasani S., Esmaeely Neisiany R., Ramakrishna S. (2019). Wound Dressings: Current Advances and Future Directions. J. Appl. Polym. Sci..

[5] Bolívar-Monsalve E.J., Alvarez M.M., Hosseini S., Espinosa-Hernandez M.A., Ceballos-González C.F., Sanchez-Dominguez M., Shin S.R., Cecen B., Hassan S., Di Maio E. (2021). Engineering Bioactive Synthetic Polymers for Biomedical Applications: A Review with Emphasis on Tissue Engineering and Controlled Release. Mater. Adv..

[6] Wieszczycka K., Staszak K., Woźniak-Budych M.J., Litowczenko J., Maciejewska B.M., Jurga S. (2021). Surface Functionalization–The Way for Advanced Applications of Smart Materials. Coord. Chem. Rev..

[7] Mi Y., Zhong L., Lu S., Hu P., Pan Y., Ma X., Yan B., Wei Z., Yang G. (2022). Quercetin Promotes Cutaneous Wound Healing in Mice through Wnt/β-Catenin Signaling Pathway. J. Ethnopharmacol..

[8] Ullah A., Munir S., Badshah S.L., Khan N., Ghani L., Poulson B.G., Emwas A.H., Jaremko M. (2020). Important Flavonoids and Their Role as a Therapeutic Agent. Molecules.

[9] Carvalho M.T.B., Araújo-Filho H.G., Barreto A.S., Quintans-Júnior L.J., Quintans J.S.S., Barreto R.S.S. (2021). Wound Healing Properties of Flavonoids: A Systematic Review Highlighting the Mechanisms of Action. Phytomedicine.

[10] Oliveira M.T.A., Teixeira A.M.R., Cassiano C.J.M., Sena D.M., Coutinho H.D.M., Menezes I.R.A., Figueredo F.G., Silva L.E., Toledo T.A., Bento R.R.F. (2016). Modulation of the Antibiotic Activity against Multidrug Resistant Strains of 4-(Phenylsulfonyl) Morpholine. Saudi J. Biol. Sci..

[11] Wang P.F., Qiu H.Y., Ma J.T., Yan X.Q., Gong H.B., Wang Z.C., Zhu H.L. (2015). Dihydropyrazoles Containing Morpholine: Design, Synthesis and Bioassay Testing as Potent Antimicrobial Agents. RSC Adv..

[12] Kumari A., Singh R.K. (2020). Morpholine as Ubiquitous Pharmacophore in Medicinal Chemistry: Deep Insight into the Structure-Activity Relationship (SAR). Bioorg. Chem..

[13] Surendra Kumar R., Moydeen M., Al-Deyab S.S., Manilal A., Idhayadhulla A. (2017). Synthesis of New Morpholine-Connected Pyrazolidine Derivatives and Their Antimicrobial, Antioxidant, and Cytotoxic Activities. Bioorg. Med. Chem. Lett..

[14] De S., Kumar S.K.A., Shah S.K., Kazi S., Sarkar N., Banerjee S., Dey S. (2022). Pyridine: The Scaffolds with Significant Clinical Diversity. RSC Adv..

[15] Ali I., Burki S., El-Haj B.M., Shafiullah, Parveen S., Nadeem H.Ş., Nadeem S., Shah M.R. (2020). Synthesis and Characterization of Pyridine-Based Organic Salts: Their Antibacterial, Antibiofilm and Wound Healing Activities. Bioorg. Chem..

[16] Allaka T.R., Katari N.K. (2022). Synthesis of Pyridine Derivatives for Diverse Biological Activity Profiles: A Review. Recent Dev. Synth. Appl. Pyridines.

[17] Kamat V., Santosh R., Poojary B., Nayak S.P., Kumar B.K., Sankaranarayanan M., Faheem, Khanapure S., Barretto D.A., Vootla S.K. (2020). Pyridine- And Thiazole-Based Hydrazides with Promising Anti-Inflammatory and Antimicrobial Activities along with Their in Silico Studies. ACS Omega.

[18] Tahir T., Ashfaq M., Saleem M., Rafiq M., Shahzad M.I., Kotwica-Mojzych K., Mojzych M. (2021). Pyridine Scaffolds, Phenols and Derivatives of Azo Moiety: Current Therapeutic Perspectives. Molecules.

[19] Yao X., Zou S., Fan S., Niu Q., Zhang Y. (2022). Bioinspired Silk Fibroin Materials: From Silk Building Blocks Extraction and Reconstruction to Advanced Biomedical Applications. Mater. Today Bio.

[20] Sabarees G., Tamilarasi G.P., Velmurugan V., Alagarsamy V., Sibuh B.Z., Sikarwar M., Taneja P., Kumar A., Gupta P.K. (2023). Emerging Trends in Silk Fibroin Based Nanofibers for Impaired Wound Healing. J. Drug Deliv. Sci. Technol..

[21] Patil P.P., Reagan M.R., Bohara R.A. (2020). Silk Fibroin and Silk-Based Biomaterial Derivatives for Ideal Wound Dressings. Int. J. Biol. Macromol..

[22] Xue J., Wu T., Dai Y., Xia Y. (2019). Electrospinning and Electrospun Nanofibers: Methods, Materials, and Applications. Chem. Rev..

[23] Gao C., Zhang L., Wang J., Jin M., Tang Q., Chen Z., Cheng Y., Yang R., Zhao G. (2021). Electrospun Nanofibers Promote Wound Healing: Theories, Techniques, and Perspectives. J. Mater. Chem. B.

[24] Zhang W., He Z., Han Y., Jiang Q., Zhan C., Zhang K., Li Z., Zhang R. (2020). Structural Design and Environmental Applications of Electrospun Nanofibers. Compos. Part A Appl. Sci. Manuf..

[25] Yang Y., Du Y., Zhang J., Zhang H., Guo B. (2022). Structural and Functional Design of Electrospun Nanofibers for Hemostasis and Wound Healing. Adv. Fiber Mater..

[26] Chouhan D., Mandal B.B. (2020). Silk Biomaterials in Wound Healing and Skin Regeneration Therapeutics: From Bench to Bedside. Acta Biomater..

[27] Kandhasamy S., Liang B., Yang D.P., Zeng Y. (2021). Antibacterial Vitamin K3 Carnosine Peptide-Laden Silk Fibroin Electrospun Fibers for Improvement of Skin Wound Healing in Diabetic Rats. ACS Appl. Bio Mater..

[28] Biagiotti M., Bassani G.A., Chiarini A., Vincoli V.T., Dal Prà I., Cosentino C., Alessandrino A., Taddei P., Freddi G. (2022). Electrospun Silk Fibroin Scaffolds for Tissue Regeneration: Chemical, Structural, and Toxicological Implications of the Formic Acid-Silk Fibroin Interaction. Front. Bioeng. Biotechnol..

[29] Chen J.P., Chen S.H., Lai G.J. (2012). Preparation and Characterization of Biomimetic Silk Fibroin/Chitosan Composite Nanofibers by Electrospinning for Osteoblasts Culture. Nanoscale Res. Lett..

[30] Selvaraj S., Fathima N.N. (2017). Fenugreek Incorporated Silk Fibroin Nanofibers—A Potential Antioxidant Scaffold for Enhanced Wound Healing. ACS Appl. Mater. Interfaces.

[31] Kim H., Che L., Ha Y., Ryu W. (2014). Mechanically-Reinforced Electrospun Composite Silk Fibroin Nanofibers Containing Hydroxyapatite Nanoparticles. Mater. Sci. Eng. C.

[32] Park Y.R., Ju H.W., Lee J.M., Kim D.-K.K., Lee O.J., Moon B.M., Park H.J., Jeong J.Y., Yeon Y.K., Park C.H. (2016). Three-Dimensional Electrospun Silk-Fibroin Nanofiber for Skin Tissue Engineering. Int. J. Biol. Macromol..

[33] Kim J.H., Park C.H., Lee O.J., Lee J.M., Kim J.W., Park Y.H., Ki C.S. (2012). Preparation and in Vivo Degradation of Controlled Biodegradability of Electrospun Silk Fibroin Nanofiber Mats. J. Biomed. Mater. Res. Part A.

[34] Lee C.H., Hsieh M.J., Chang S.H., Lin Y.H., Liu S.J., Lin T.Y., Hung K.C., Pang J.H.S., Juang J.H. (2014). Enhancement of Diabetic Wound Repair Using Biodegradable Nanofibrous Metformin-Eluting Membranes: In Vitro and in Vivo. ACS Appl. Mater. Interfaces.

[35] Govindarajan D., Lakra R., Korapatti P.S., Ramasamy J., Kiran M.S. (2019). Nanoscaled Biodegradable Metal-Polymeric Three-Dimensional Framework for Endothelial Cell Patterning and Sustained Angiogenesis. ACS Biomater. Sci. Eng..

[36] Homaeigohar S., Boccaccini A.R. (2020). Antibacterial Biohybrid Nanofibers for Wound Dressings. Acta Biomater..

[37] Chittasupho C., Manthaisong A., Okonogi S., Tadtong S., Samee W. (2022). Effects of Quercetin and Curcumin Combination on Antibacterial, Antioxidant, in Vitro Wound Healing and Migration of Human Dermal Fibroblast Cells. Int. J. Mol. Sci..

[38] Sunil Richard A., Verma R.S. (2022). Antioxidant α-Mangostin Coated Woven Polycaprolactone Nanofibrous Yarn Scaffold for Cardiac Tissue Repair. ACS Appl. Nano Mater..

[39] Yunus Basha R., Sampath Kumar T.S., Selvaraj R., Doble M. (2018). Silver Loaded Nanofibrous Curdlan Mat for Diabetic Wound Healing: An In Vitro and In Vivo Study. Macromol. Mater. Eng..

[40] Kandhasamy S., Zeng Y. (2022). Fabrication of Vitamin K3-Carnosine Peptide-Loaded Spun Silk Fibroin Fibers/Collagen Bi-Layered Architecture for Bronchopleural Fistula Tissue Repair and Regeneration Applications. Biomater. Adv..

[41] Agarwal Y., Rajinikanth P.S.S., Ranjan S., Tiwari U., Balasubramnaiam J., Pandey P., Arya D.K., Anand S., Deepak P. (2021). Curcumin Loaded Polycaprolactone-/Polyvinyl Alcohol-Silk Fibroin Based Electrospun Nanofibrous Mat for Rapid Healing of Diabetic Wound: An in-Vitro and in-Vivo Studies. Int. J. Biol. Macromol..

[42] Wang Q., Zhou S., Wang L., You R., Yan S., Zhang Q., Li M. (2021). Bioactive Silk Fibroin Scaffold with Nanoarchitecture for Wound Healing. Compos. Part B Eng..

[43] Shefa A.A., Sultana T., Park M.K., Lee S.Y., Gwon J.G., Lee B.T. (2020). Curcumin Incorporation into an Oxidized Cellulose Nanofiber-Polyvinyl Alcohol Hydrogel System Promotes Wound Healing. Mater. Des..

[44] Ahn S., Chantre C.O., Gannon A.R., Lind J.U., Campbell P.H., Grevesse T., O’Connor B.B., Parker K.K. (2018). Soy Protein/Cellulose Nanofiber Scaffolds Mimicking Skin Extracellular Matrix for Enhanced Wound Healing. Adv. Healthc. Mater..

[45] Guo X., Xia B., Lu X.B., Zhang Z.J., Li Z., Li W.L., Xiong A.B., Deng L., Tan M.Y., Huang Y.C. (2016). Grafting of Mesenchymal Stem Cell-Seeded Small Intestinal Submucosa to Repair the Deep Partial-Thickness Burns. Connect. Tissue Res..

