# Supplementary material for: Fabrication of Quercetin-Functionalized Morpholine and Pyridine Motifs-Laden Silk Fibroin Nanofibers for Effective Wound Healing in Preclinical Study
Source: Pharmaceutics. 2024 Mar 26;16(4):462. doi: 10.3390/pharmaceutics16040462 (PMC11054860; doi:10.3390/pharmaceutics16040462)
Supplement: Supplementary file 1 [file pharmaceutics-16-00462-s001.zip › pharmaceutics-2888139-supplementary.pdf]

Supporting Information

Fabrication of Quercetin-Functionalized Morpholine and Pyridine Motifs-Laden Silk Fibroin Nanofibers for Effective Wound Healing in Preclinical Study

Govindaraj Sabarees[1], Vadivel Velmurugan[1*], Siddan Gouthaman[2], Viswas Raja Solomon [3*] and Subramani Kandhasamy[3*]

[1]Department of Pharmaceutical Chemistry, SRM College of Pharmacy, SRM Institute of Science and Technology, Kattankulathur, Chennai 603203, India

[2]Organic Material Laboratory, Department of Chemistry, Indian Institute of Technology, Roorkee 247667, India

[3]Medicinal Chemistry Research Laboratory, MNR College of Pharmacy, Gr. Hyderabad, Sangareddy 502294, Andhra Pradesh, India

[4]School of Mechanical and Electrical Engineering, Quanzhou University of Information Engineering, Quanzhou, Fujian 362000, China.

Correspondence

*E-mail: velmuruv@srmist.edu.in
**E-mail: kandhasks@gmail.com
***vrajasolomon@gmail.com

Page No

1. General procedure for synthesis of quercetin pyridyl hydrazone 3a (QFM): S-2
2. NMR, FTIR, ESI-MS measurement :S-2
3. **Figure S1** ^{1}H NMR spectrum :S-3
4. **Figure S2** ^{13}C NMR spectrum :S-3
5. **Figure S3** ESI-MS spectrum :S-4
6. **Figure S4** Results of the in vitro cytotoxicity (MTT) assay for Quercetin. :S-4

1. *General procedure for the synthesis of quercetin pyridyl hydrazone **3a** (QFM):*

The quercetin dihydrate (**aa**) (2.0 g, 6.63 mmol, 11.0 equiv), was dissolved in 20 mL of methanol heated at 60 °C for 1 h in a separate flask. The solution underwent treatment using 2-morpholinoethan-1-amine (**ac**) (2.0 g, 7.95 mmol, 1.2 equiv) in combination with 0.4 g of paraformaldehyde (**ab**) at a ratio of 5:1 by weight. The reaction mixture was subjected to a thermal treatment at 60 °C for 15 min. Subsequently, the solution **aa** was introduced into the reaction mixture. The reaction mixture was subjected to a 4 h extension at a temperature of 60 °C. After the reaction, optimization was performed using thin-layer chromatography (TLC). Subsequently, the reaction mixture was allowed to cool to ambient temperature. Subsequently, the yellow solid residue was collected by the process of filtration. Later, the material underwent a refinement process by crystallization in methanol that was heated, resulting in a yield of 74% (2.20 g) of **1a**. A solution containing 20 mL of methanol dissolved 0.5 g of compound **1a** (0.5 g, 1.12 mmol, 1.0 equiv), and a one-hour heating period was applied to the resulting mixture. Afterward, reactant **2a** (0.14 g, 1.24 mmol, 1.1 equiv.) was added to the reaction mixture, allowing the reaction to continue for another four hours. After the reaction had concluded, the reaction mixture was allowed to reach room temperature. Through filtration, the intense yellow solid residue was subsequently collected. The substance was then refined by crystallization in heated methanol, resulting in a **3a** (0.5 g, 83%) yield.

2. *NMR, FTIR, ESI-MS measurement*

Bruker 400 MHz was employed for the purpose of conducting nuclear magnetic resonance (NMR) spectroscopy. In order to monitor the solubility of the compounds, a deuterated NMR solvent was utilized to record the NMR spectra. Specifically, CDCl3 was employed to acquire both ^{1}H NMR and 13C NMR spectra. The values for chemical alterations were reported in parts per million (ppm). The reference resonance peaks were established at 7.26 ppm ($CHCl_3$) and 2.50 ppm [$(CD_2H)_2SO$] for ^{1}H NMR spectra and 77.23 ppm ($CDCl_3$) and 39.52 ppm (DMSO-d_6), for ^{13}C NMR spectra. Chemical shifts from tetramethylsilane are reported in ppm downfield and coupling constants are reported in Hz. ATR-FTIR spectroscopy (SHIMADZU, IRTRACER 100) was used to analyze the functional group of all synthesized QFM compound SF and SF-QFM nanofibrous mats within a frequency range of 4000-600 cm^{-1}.

The mass of the synthesized QFM compound was determined using Electrospray ionization mass spectrometry (ESI-MS), which detected the mass peaks $[M+Na]^{+}$.

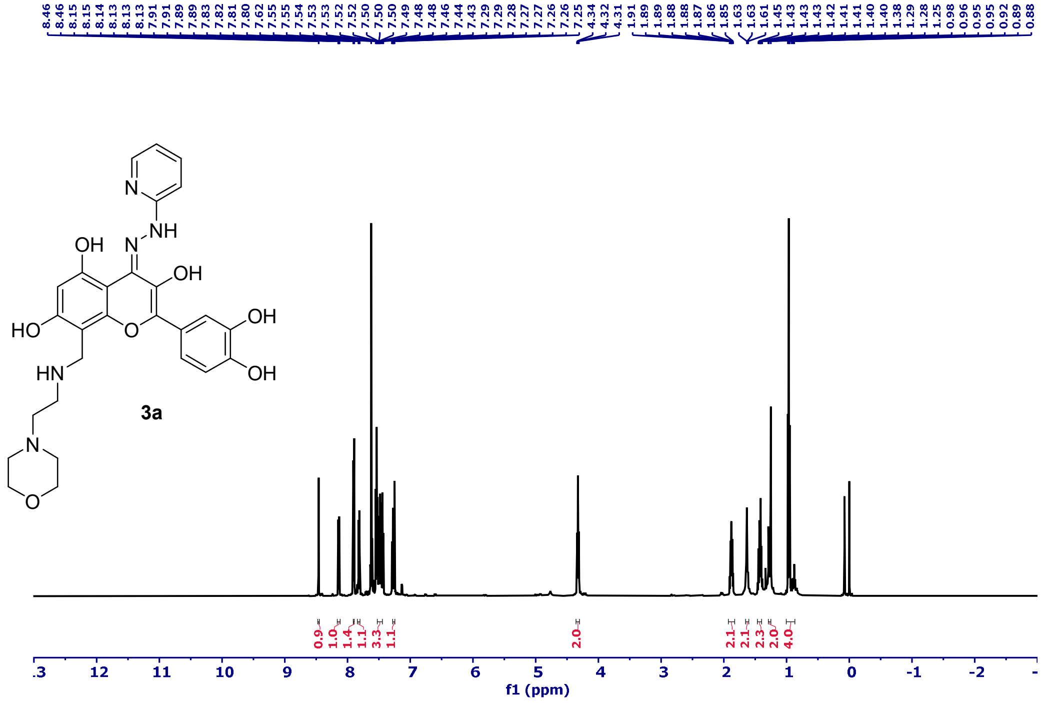

Figure S1. ^{1}H NMR Spectrum of compound **3a**

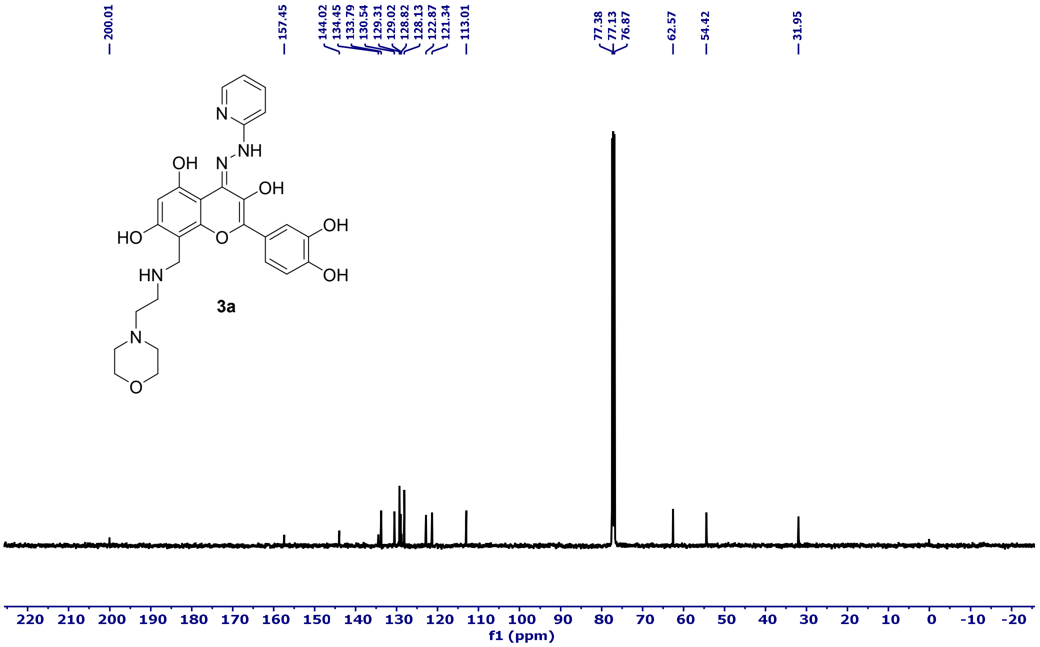

Figure S2. ^{13}C NMR Spectrum of compound **3a**

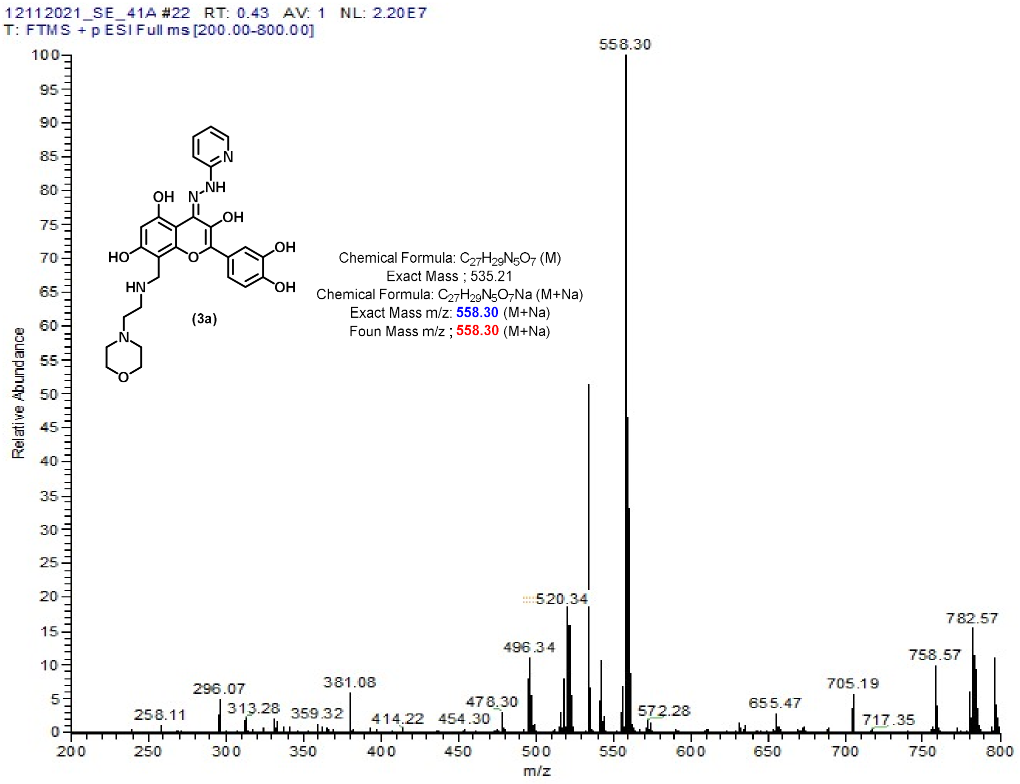

Figure S3. ESI-MS spectrum for compound **3a**

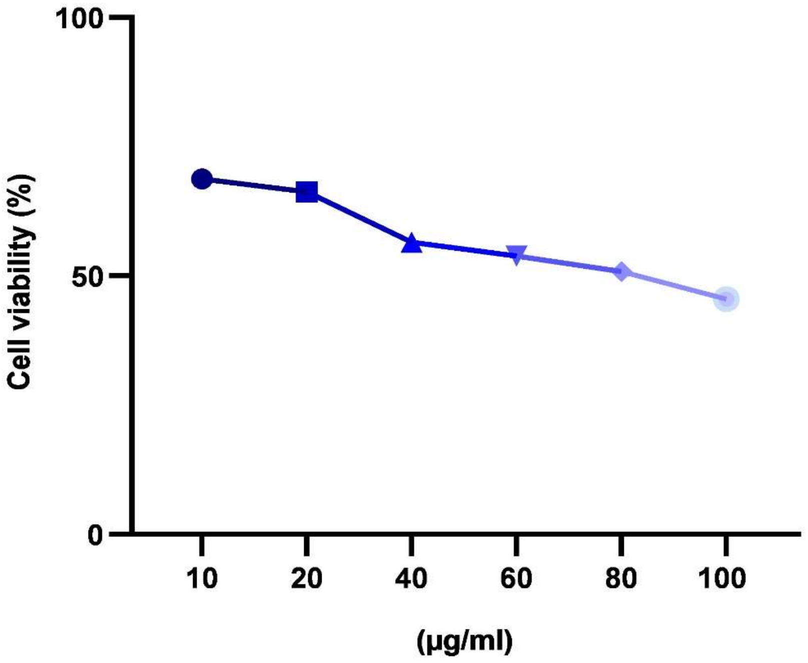

Figure S4. Results of the in vitro cytotoxicity (MTT) assay for Quercetin.
